# A Piano Training Program to Improve Manual Dexterity and Upper Extremity Function in Chronic Stroke Survivors

**DOI:** 10.3389/fnhum.2014.00662

**Published:** 2014-08-22

**Authors:** Myriam Villeneuve, Virginia Penhune, Anouk Lamontagne

**Affiliations:** ^1^School of Physical and Occupational Therapy, McGill University, Montreal, QC, Canada; ^2^Feil and Oberfeld Research Centre, Jewish Rehabilitation Hospital, Research Site of the Montreal Center for Interdisciplinary Research in Rehabilitation (CRIR), Laval, QC, Canada; ^3^Laboratory for Motor Learning and Neural Plasticity, Department of Psychology, Concordia University, Montreal, QC, Canada

**Keywords:** cerebrovascular accident, hand, paresis, learning, music, rehabilitation

## Abstract

**Objective:** Music-supported therapy was shown to induce improvements in motor skills in stroke survivors. Whether all stroke individuals respond similarly to the intervention and whether gains can be maintained over time remain unknown. We estimated the immediate and retention effects of a piano training program on upper extremity function in persons with chronic stroke.

**Methods:** Thirteen stroke participants engaged in a 3-week piano training comprising supervised sessions (9 × 60 min) and home practice. Fine and gross manual dexterity, movement coordination, and functional use of the upper extremity were assessed at baseline, pre-intervention, post-intervention, and at a 3-week follow-up.

**Results:** Significant improvements were observed for all outcomes at post-intervention and follow-up compared to pre-intervention scores. Larger magnitudes of change in manual dexterity and functional use of the upper extremity were associated with higher initial levels of motor recovery.

**Conclusion:** Piano training can result in sustainable improvements in upper extremity function in chronic stroke survivors. Individuals with a higher initial level of motor recovery at baseline appear to benefit the most from this intervention.

## Introduction

Most stroke survivors experience upper extremity impairments (Hendricks et al., 2002) that can result in persistent activity and participation limitations. Existing approaches for upper extremity rehabilitation have been shown to yield modest to moderate improvements (Van Peppen et al., 2004), possibly due to insufficient training intensity and treatment adherence. Current literature on motor learning and recovery indicates that interventions should be meaningful, task-specific, tailored to the person’s capacity and interests, and provide sufficient repetition and challenge to induce training effects (Van Peppen et al., 2004; Hubbard et al., 2009). Rehabilitation interventions can further take advantage of multi-sensory feedback to provide knowledge of results and/or performance (Cirstea and Levin, 2007).

Music-supported therapy (MST) uses a music-learning paradigm to support motor rehabilitation. It is hypothesized that auditory feedback may facilitate learning and performance and that the musical context makes the therapy more engaging and rewarding as compared to conventional approaches. MST was shown to yield improvements in manual dexterity in both acute and chronic stroke survivors (Altenmuller et al., 2009; Amengual et al., 2013). Electrophysiological measures further demonstrated that MST may build on auditory–motor coupling mechanisms to drive cortical facilitation and brain plasticity (Amengual et al., 2013). Despite the potential of MST for upper extremity rehabilitation, previous studies have not tested whether gains can be maintained on the longer-term. Furthermore, as stroke survivors present with a range of severity, there is a need to determine who best respond to this intervention. Finally, existing MST programs consist of mixed-instrument protocols (piano and drum pads) that require daily supervised sessions (Altenmuller et al., 2009; Amengual et al., 2013). Such resource intensive protocols may be difficult to implement in the clinical setting or at home. Existing protocols also lack details on training parameters and criteria for progression.

In the present study, we have developed an individually tailored piano training program that combines structured and supervised training sessions with home practice. The specific objectives of this study were to estimate the immediate and retention effects of a 3-week piano training program on manual dexterity, finger movement coordination, and functional use of upper extremity in chronic stroke survivors and to establish the relationship between the participants’ characteristics and intervention outcomes. We hypothesized that MST improves upper extremity function and piano-related outcomes. We also hypothesized that participants may respond differently to the intervention depending on their clinical profile, including their age, chronicity, and initial level of motor recovery.

## Materials and Methods

### Participants

A convenience sample of 13 chronic stroke survivors was recruited among discharged patients of 2 rehabilitation centers in the Montreal area. Inclusion criteria were: (1) first supratentorial chronic stroke (>6 months) in the middle cerebral artery territory confirmed by CT scan or magnetic resonance imaging; (2) a motor deficit of the paretic upper extremity but some capacity for active wrist and finger movements [scores of 3–6 out of 7 on the arm and hand components of the Chedoke McMaster Stroke Assessment (CMSA)] (Gowland et al., 1993; Hubbard et al., 2009) and; (3) corrected to normal vision. Participants were excluded if having moderate to severe cognitive deficits (scores ≤23 on the Montreal Cognitive Assessment) (Nasreddine et al., 2005), visual field defect (Goldmann perimetry), or visuospatial neglect (Bell’s test), if still receiving therapy for the upper extremity or if having another condition interfering with upper extremity movements. Individuals with professional musical experience and/or more than 1 h per week of practice of any musical instrument during the past 10 years were not included in the study. Note that two participants were found *a posteriori* to have a lesion that involved the brainstem and the cerebellum. The study was approved by the Institutional Ethics Committee and written informed consent was obtained from each participant.

### General procedure

Participants were assessed on clinical outcomes at baseline (week_0_), pre-intervention (week_3_), post-intervention (week_6_), and at follow-up (week_9_). Training sessions and evaluations were performed by the same therapist. The intervention consisted of three individual 1-h sessions per week for three consecutive weeks for a total of nine sessions. Piano performance measures were collected at every session. Supervised sessions were complemented with a biweekly home program (30 min/session).

**Figure 1:**
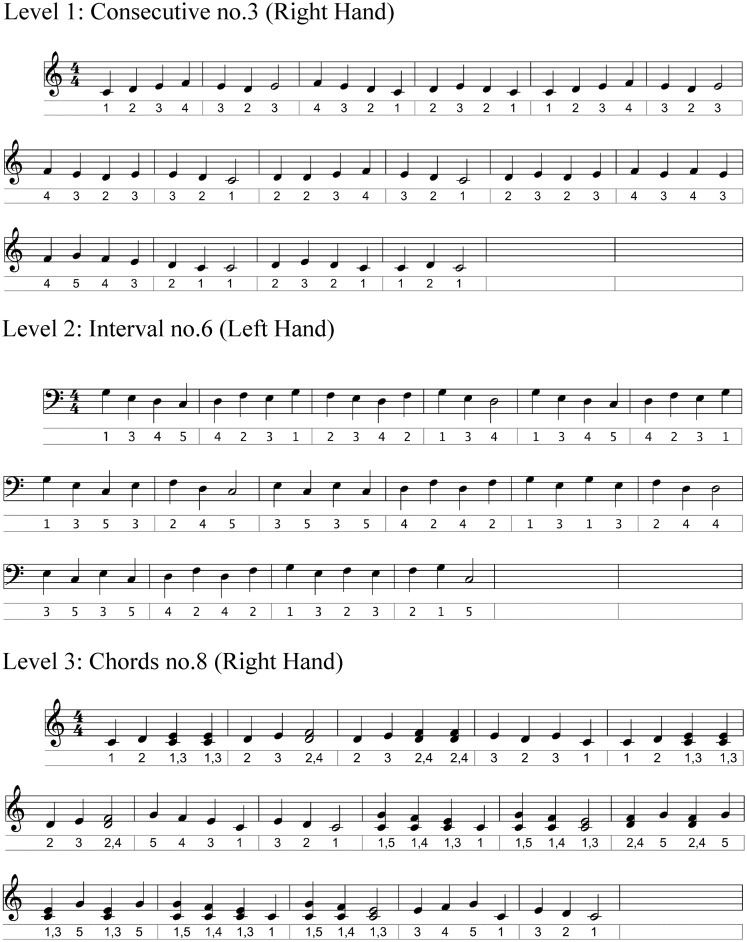
**Excerpts of musical scores of three pieces, one at each level, with digit number under each note**.

**Figure 2:**
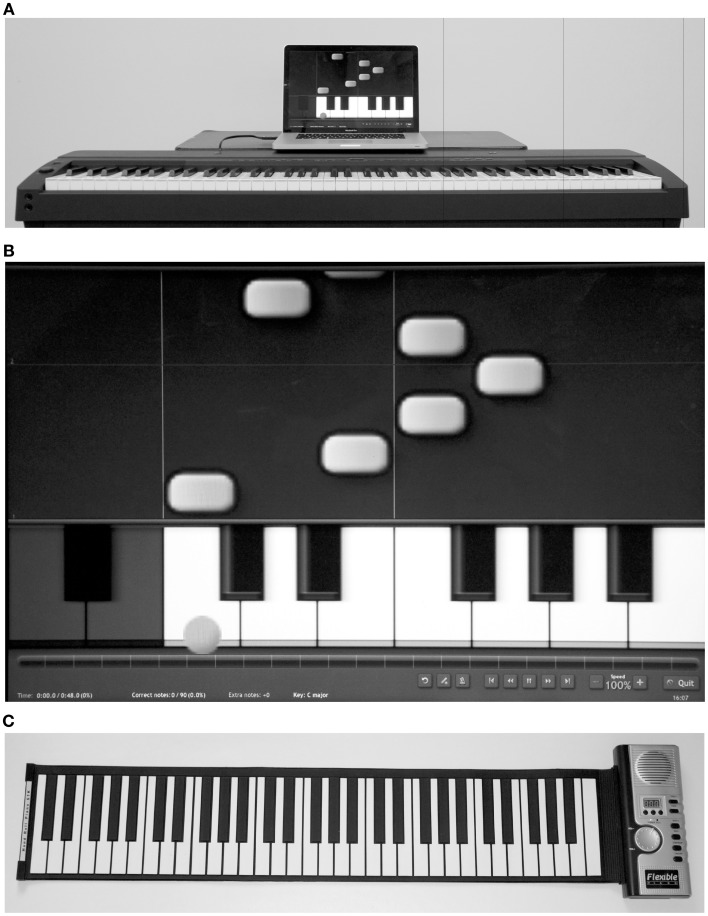
**(A)** Structured training session setting; **(B)** screen shot of Synthesia Musical Instrument Digital Interface (MIDI) piano program; **(C)** Roll-up flexible piano.

Musical pieces, created with Harmony Assistant™(Myriad, Toulouse), involved all five digits of the paretic hand. Whether played with the right or left hand, they involved the same number of finger repetitions and similar finger sequences. Pieces were composed by an experienced musician and were designed to be musically pleasant based on simple harmonic rules of composition as well as of relatively short duration and easy to remember (Figure 1). Musical pieces were displayed with Synthesia™(Synthesia LLC), a software program adapted for people with no music reading abilities. A visual display cued the sequence of key presses required to produce each melody by showing a blue dot falling from the upper part of the screen down to the correct key on a virtual keyboard (Figure 2). After each cue, the program paused until the participant pressed the correct key before moving on. During the supervised training sessions, participants played on a touch sensitive Yamaha P-155™ piano keyboard (Yamaha). They received feedback on their performance through Synthesia and through the therapist who provided verbal feedback on the quality of movement and compensatory strategies. Home piano exercises were executed on a roll-up flexible piano (Hand Roll Piano, 61K™), without Synthesia.

### Intervention

Nine musical pieces were introduced in an order of increasing difficulty: *level 1* involved movements of consecutive fingers [e.g., digit 1–2–3–4–5]; *level 2* involved third, fourth, and fifth intervals or movements of non-consecutive fingers [e.g., 1–3–5–2–4] and; *level 3* involved chords, that is two fingers played at the same time. Within each level, three musical pieces that involved an increasing number of key presses and changes in melodic direction were introduced (Table 1). In addition, the speed of execution or tempo increased within each musical piece: participants started at a tempo of 30 beats per minute (bpm) and once reaching ≥80% accuracy [1−(#errors/#key presses) × 100] on three consecutive trials, the tempo was increased by steps of 10% until reaching 60 bpm. After the latter tempo was reached, the next musical piece was introduced. During the home practice sessions, participants were asked to reproduce short digit sequences on the roll-up piano. These sequences comprised short excerpts of the same musical pieces practiced during the supervised sessions and consisted of 30 written exercises where all 5 fingers were represented as a number (1 = thumb, 5 = pinky). Participants reported on their practice duration and content in a logbook after each practice session.

**Table 1:** **Musical piece’s progression**.

Song #	Piece duration (s)^a^	Number of notes	Number of changes in melodic direction
**LEVEL 1: CONSECUTIVE NOTES**			
1	32	52	11
2	38	69	32
3	48	87	38
**LEVEL 2: INTERVALS**			
4	32	40	18
5	32	59	34
6	48	90	40
**LEVEL 3: CHORDS**			
7	32	53	16
8	32	82	26
9	48	136	31

*^a^Piece duration at 60 bpm*.

### Outcome measures

Piano performance measures included the number of errors (incorrect or early key presses) and duration of the musical pieces recorded with Synthesia as well as the total number of pieces completed after the nine sessions. The following clinical measures were also collected at baseline, pre- and post-intervention, and follow-up. The Box and Block Test (BBT) and Nine Hole Peg Test (NHPT) were used to evaluate gross and fine manual dexterity, respectively. The functional use of the upper extremity was assessed with the six-item version of the Jebsen Hand Function Test (Jebsen). The Finger to Nose Test (FTN) and Finger Tapping Test (FTT) were chosen as measures of arm and finger movement coordination, respectively.

At post-intervention, feedback was collected using a custom-designed questionnaire. The questionnaire included questions where participants rated their interest in the structured piano sessions, the home practice exercises, and the musical pieces using a numerical rating scale (score of 0 = not interesting and 10 = very interesting). Open-ended questions further investigated whether participants had experienced adverse or undesirable effects during the intervention, and whether they had observed changes in upper extremity function after the training. Any additional written and verbal comments were collected.

### Data and statistical analysis

We conducted a linear mixed model analysis for repeated measures with autoregressive covariance structure, while controlling for baseline measurements (week_0_), with time [pre (week_3_), post (week_6_), and follow-up (week_9_)] as a within-subject factor to assess the effect of the intervention on the clinical measures. *Post hoc* pairwise comparisons were used to identify differences between measurement time-points. Correlations were carried out between change scores on the clinical measures and characteristics of the participants at baseline [age, time since stroke, motor recovery (CMSA), and manual dexterity (BBT)]. Pearson correlation coefficients were used for all outcomes, except for the level of motor recovery for which Spearman’s rank correlation coefficients were used. Statistical analyses were performed in SPSS V20. The level of significance was set to *p* < 0.05. For each family of outcome measures, we controlled for family-wise error using modified Bonferroni correction.

## Results

Participants’ characteristics are presented in Table 2. Based on the arm and hand components of the CMSA, participants were classified as mildly affected (score of 6), moderately affected (scores of 4 or 5), or severely affected (score of 3). Seven participants suffered from a subcortical stroke, four had a cortical stroke, while two participants were found post-priori to have pontine or cerebellar lesions. Six participants had a right hemisphere lesion, six had a left lesion, and one participant showed bilateral lesions. Out of the 13 participants, only 5 had prior musical training, which included 1–5 years of non-professional piano experience before the age of 18, with the exception of one participant (#11) who played occasionally (<1 h/week) in the 2 years preceding stroke onset but had no formal musical training. Participants were free of cognitive deficits as indicated by MoCA scores ranging from 28 to 30. All were living in the community and average school attendance was 14 ± 3.7 years (mean ± 1 SD).

**Table 2:** **Participant characteristics at baseline**.

Participant	Age	Gender	Lesion localization	Etiology	Time since stroke	CMSA arm/hand	Spasticity (MAS)	Musical experience	MoCA score
1	62	F	Right basal ganglia	I	118	3/3	3	5	30
2	71	F	Left pontine medullary	I	112	3/3	3	0	30
3	52	M	Right basal ganglia	I	40	3/3	3	1	30
4	54	M	Bilateral cerebellum (L > R) and left thalamus	I	14	4/4	0	0	30
5	49	M	Left sub-arachnoids and left sylvian fissure	H	32	5/4	1	0	30
6	41	M	Right frontal cortex, right basal ganglia, right head of caudate, and right corona radiata	I	44	5/4	2	0	30
7	75	M	Left fronto-parietal region	I	18	6/6	0	0	30
8	75	M	Right thalamus and internal capsule	H	6	6/6	0	0	29
9	74	M	Left thalamus	I	12	6/6	0	0	29
10	79	F	Right sylvian para-central gyrus	I	15	6/6	0	1	30
11	60	F	Right intraparenchymal frontal region	H	61	6/6	0	2	30
12	32	F	Left intraventricular and left thalamus	H	16	6/6	0	1	28
13	57	M	Left posterior limb of internal capsule	I	64	6/6	0	0	30

*Age (years), gender (male/female), etiology (hemorrhagic/ischemic), time since stroke (months), CMSA = Chedoke McMaster Stroke Assessment (arm/hand scores, max = 7), MAS = Modified Ashworth Scale (max = 5), musical experience (years), MoCA = Montreal Cognitive Assessment Test (max = 30)*.

All participants completed the nine training sessions over 3 weeks, except participants #3 and #6 who, due to personal constraints, completed the program over 4 weeks. Two to nine musical pieces were completed, with some participants reaching *level 1* (*n * = 4) and others reaching *level 2* (*n* = 5) and *level 3* (*n* = 4) at post-intervention (Table 3). Each piece was practiced on average 25 times before reaching 80% accuracy at 60 bpm. At 60 bpm, average durations of musical pieces ranged from 32 to 48 s. Mean home practice time was 28 min/session, with seven participants meeting/exceeding practice time requirement. All participants performed the home exercises independently.

**Table 3:** **Changes on motor function tests post- vs. pre-intervention**.

Participants	BBT		NHPT		FTN		Index FTT		Jebsen		Home Practice	Training progression
	Δ	%	Δ	%	Δ	%	Δ	%	Δ	%	Time (min)	# songs/level
**SEVERELY AFFECTED**												
1	4	200	Ø	Ø	5	100.0	5	100.0	Ø	Ø	170	2/1
2	4	33.3	Ø	Ø	Ø	Ø	6	100.0	−60.9	−12.1	180^c^	2/1
3	7^a^	50	Ø	Ø	5	55.6	5	50.0	−63.2	−31.0	185^c^	2/1
**MODERATELY AFFECTED**												
4	3	25	Ø	Ø	1	14.3	8	47.1	−63.8	−31.5	50	5/2
5	6^a^	27.3	−17.7	−15.0	4	36.4	4	33.3	−61.9	−44.5	60	7/3
6	4	14.3	−36.6^a^	−29.6	7	58.3	2	9.1	−39.7	−43.3	135	5/2
**MILDLY AFFECTED**												
7	11^a^	30.6	−19.4	−40.3	4	25.0	1	3.6	−17.5^b^	−27.6	227^c^	5/2
8	6^a^	14.6	−11.9^b^	−32.2	5	31.3	2	6.3	−26.5	−31.8	155	5/2
9	10^a^	28.6	−7.4	−14.0	5	29.4	4	9.8	−9.5^b^	−18.7	140	6/2
10	7^a^	16.3	−9.6^b^	−28.3	5	35.7	2	3.6	−12.1^b^	−25.4	195^c^	6/2
11	5^a^	9.3	−7.1^b^	−25.8	6	23.8	6	9.4	−20.0^b^	−41.6	245^c^	9/3
12	12^a^^b^	21.1	−7.1^b^	−29.2	3	12.5	21	46.7	−3.0^b^	−11.6	225^c^	9/3
13	17^a^^b^	32.1	−2.7^a^^b^	−11.1	7	36.8	7	17.1	−10.6^a^	−31.1	315^c^	8/3
**Mean (sd)**	7.4^a^ (4.1)	38.6 (49.6)	−13.3 (10.2)	−25.1 (9.7)	4.7 (1.6)	35.3 (25.2)	5.6 (5.1)	33.5 (34.2)	−32.4 (24.0)	−27.1 (12.9)	175.5 (72.1)	5.5 (2.4)

Δ, Change between pre- and post-intervention; %, percent change between pre- and post-intervention; Ø, participant unable to perform the test;

^a^participant reached the smallest real difference (SRD) score;

^b^participant reached the norms for his/her age group;

*^c^Participant practiced at least 180 min at home (2 min × 30 min)*.

Participants completed all the evaluations. Some, however, were unable to complete specific tests [NHPT (*n* = 4), FTN (*n* = 1), and Jebsen (*n* = 1)] at any of the evaluation time-points due to their low level of motor recovery. No significant differences were found between baseline and pre-intervention scores for any of the clinical tests, including the BBT, NHPT, FTN, FTT, and Jebsen (*t*-tests, *p* > 0.05). The linear mixed model showed significant effects of the intervention (*p* < 0.0001) on the BBT [*F*(2,24) = 38.70], NHPT [*F*(2,16) = 17.50], FTN [*F*(2,22) = 101.59], FTT [*F*(2,24) = 14.74], and the Jebsen [*F*(2,21) = 24.02]. *Post hoc* analyses revealed that scores for all clinical outcomes were significantly higher at post-intervention compared to pre-intervention (*p* < 0.0001), while there was no significant difference between post-intervention and follow-up (*p* > 0.5).

**Figure 3:**
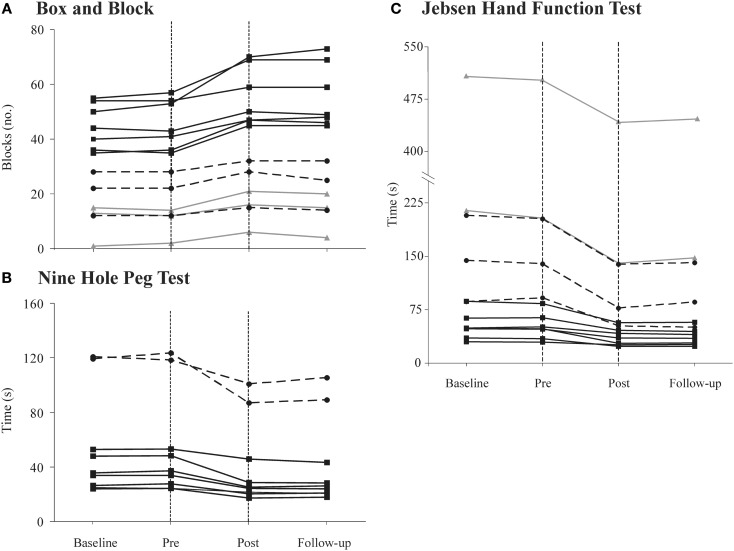
Individual performances for all participants [severely affected (gray solid line), moderately affected (black dashed line), and mildly affected (black solid line)] on the **(A)** Box and Block Test (BBT); **(B)** Nine Hole Peg Test (NHPT) and; **(C)** Jebsen Hand Function Test (Jebsen) at baseline, pre-intervention, post-intervention, and follow-up. The area between the vertical doted lines represents the 3-week intervention period. In **(C)**, the *y*-axis is discontinued for a better overview of results. Note that four participants could not complete the NHPT and one could not complete the Jebsen (see text for more details).

Although every participant showed improvements on all clinical tests, a large variability in initial scores as well as change scores was observed across participants (Table 3; Figure 3). In general, larger changes on the BBT were observed in the mildly affected participants, whereas larger changes on the NHPT and Jebsen were seen in the moderately and/or severely affected participants. Among the seven participants classified as “mildly affected,” many scored within the norms (mean ± 1 SD) at post-intervention on the BBT (*n* = 2), NHPT (*n* = 5), and on all subtests of the Jebsen (*n* = 6) (see norms, Mathiowetz et al., 1985; Hackel et al., 1992; Oxford Grice et al., 2003). None of these participants scored within the norms prior to the intervention.

No significant relationships were observed between change scores on the clinical measures as a result of the intervention (absolute changes between post vs. pre-intervention on the BBT, NHPT, FTN, FTT, and Jebsen) and variables such as age and time since stroke (*r* = 0.01–0.27, *p* ≥ 0.3). Participants with larger baseline scores on the BBT showed larger change scores on the BBT and Jebsen (*r* = 0.64–0.70, *p* < 0.01), but not on other clinical tests (NHPT, FTN, and FTT, *p* > 0.1). Participants with higher scores on the hand component of CMSA also showed larger change scores on the BBT (*r* = 0.54, *p* < 0.05) and the Jebsen (*r* = 0.63, *p* < 0.01), but no significant correlations were observed for other clinical tests (*p* > 0.5). Better scores on the BBT, NHPT, FTN, and FTT at baseline were associated with longer home practice durations (*r* = 0.5–0.7, *p* > 0.05).

In response to the feedback questionnaire, participants rated their interest in the supervised training session between 8 and 10, while their interest in the musical pieces ranged between 7 and 9. Their interest in the home program received scores that ranged between 2 and 10. Answers to the open-ended questions revealed that five participants considered the home training to be less interesting and not as motivating compared to the supervised sessions due to the lack of feedback received during playing. Six participants reported that the training was good for their mood and motivation to engage in upper extremity exercises, and 11 participants reported that they observed a change in upper extremity function, expressed as an increased mobility of their paretic hand, improvement in fluidity of movements as well as increased coordination and dexterity. More concretely, three participants mentioned that they could pick up small objects more easily and that they dropped objects less often when using the paretic hand, while two participants reported improvements in writing and typing skills. Three participants reported adverse or negative effects, including shoulder stiffness (*n* = 1), fatigue (*n* = 1), and mild hand numbness (*n* = 1), which resolved either immediately or within the hour following the session. Finally, five participants expressed the desire to continue piano lessons after their participation in the study; reasons mentioned included the sense of achievement and success (*n* = 2) and the perceived change in motor function and desire to experience further recovery (*n* = 3).

## Discussion

This study examined, for the first time, the short-term and retention effects of a 3-week piano training program that included supervised sessions and home practice. For this purpose, we have developed a structured program with graded levels and clear criteria for progression, which is amenable to use in clinical setting by rehabilitation therapists who do not have specialized musical training. Gains in upper extremity function were observed in all participants, with larger improvements being observed in those with higher levels of motor recovery at baseline. Gains were maintained 3 weeks after the end of intervention, suggesting that the intervention results in longer-term improvements in upper extremity function.

Our results are consistent with previous research in acute stroke where improvements in finger movement coordination and manual dexterity were reported as a result of a 3-week mixed-instrument intervention (Altenmuller et al., 2009). The fact that fine and gross manual dexterity was improved in chronic stroke survivors in the context of our study may be attributable to the specificity of the piano intervention, which involved repeated practice of dissociated finger movements while promoting speed and movement accuracy. Other contributing factors include the rapid establishment of auditory–motor coactivation induced by musical training (Altenmuller et al., 2009; Amengual et al., 2013; Grau-Sanchez et al., 2013) and the melody that is a powerful source of auditory feedback providing instantaneous knowledge of movement timing and accuracy. Hence, both the temporal and spatial features of finger movements can be trained, leading to enhanced movement coordination.

In the present study, participants also experienced an enhancement in the functional use of the paretic upper extremity as a result of the intervention, indicating that gains were transferred to functional tasks of daily living and that finger movement coordination training could be a key component of upper extremity rehabilitation. The piano intervention, which yielded a mean increase of 7.4 blocks on the BBT, may compare advantageously with other therapies such as constraint-induced movement therapy where gains of 4–4.5 blocks were reported after intensive arm restriction (3 h to 90% of waking hours for 2–4 weeks) (Leung et al., 2009; Siebers et al., 2010). Since the piano intervention relies on user-friendly and commercially available equipment, it also has the potential to be self-managed and pursued beyond the usual rehabilitation time frame.

A large variability in terms of initial scores and change scores on the different clinical outcomes was observed across participants. Given this variability, individual responses were examined with regard to smallest real differences, which are available for the BBT (+6 blocks) and the NHPT (−32.8 s) (Chen et al., 2009); eight participants exceeded the smallest real difference for the BBT (Table 3). Although only one participant reached the smallest real difference for the NHPT, it was observed that five participants (mildly affected) scored within norms at post-intervention. Similarly, six mildly affected participants attained the norms on the Jebsen. Participants with higher scores on the CMSA and BBT at baseline were also the ones who showed larger gains on most outcome measures, along with longer home practice durations. It cannot be excluded, however, that some of the clinical tests used in this study might not be sensitive enough to detect changes in individuals with more severe deficits in motor recovery. In fact, the NHPT proved to be too difficult to use in four participants who were severely or moderately affected, such that changes in fine manual dexterity that might have occurred in these individuals could not be assessed. Changes in FTN and FFT scores, however, reveal that these same participants improved in finger and arm movement coordination, in many instances to an extent that was comparable to changes observed in mildly affected participants. Taken together, these observations suggest that a significant proportion of the participants showed a true change in manual dexterity and upper extremity, and that the piano intervention has the potential to allow participants with mild impairments in motor function to improve their performance up to a level that is within normal limits. While mildly affected participants showed the largest improvements as a result of the intervention, results also show that all participants were able to complete the program, suggesting that a piano-specific intervention is feasible for chronic stroke survivors with different levels of motor recovery, including patients who only present some capacity for active wrist and finger movements (CMSA score of 3) (Gowland et al., 1993).

The location (cortical vs. subcortical) and side of stroke are often important factors to consider in assessing intervention outcomes. Participants investigated in this study predominantly suffered from subcortical stroke, with an equal distribution of left- and right-sided lesions. It was not possible to conduct statistical analysis on both subgroups due to the small number of participants. However, we can observe that six out of seven participants presenting a subcortical lesion reached the smallest real difference on the BBT, as did three out of four in the cortical subgroup. Similarly, we can observe that five out of six participants with a left lesion reached the smallest real difference on the BBT, as compared to four out of six participants with a right lesion. Although these observations do not suggest a clear difference between the type and side of the lesion, further investigations are needed with a larger sample size to validate these observations.

Information from the participant feedback questionnaire indicated that participants enjoyed the training program and felt motivated, especially during the supervised training sessions. All participants were able to complete the 60-min sessions while keeping a high level of motivation and attention for the entire practice time. We believe that the music-like nature of the pieces was an important factor that motivated participants to engage in training that requires many repetitions. We also believe that the “gaming” nature of the training (with scores and levels) added a sense of success and an awareness of improvement that made the intervention gratifying. Some participants rated their satisfaction with the home practice as lower due to the absence of feedback. However, most met or exceeded the requested practice time. Some participants even expressed the desire to pursue the piano lessons after the intervention. The sense of achievement and success, the perception of being engaged in a socially valued leisure activity, and the observation of improvements in upper extremity function are factors that may encourage stroke survivors to continue piano training on the long term, such that gains can be maintained and possibly further improved. Although MST should involve minimal risks or disadvantages, these had never been reported in previous studies. In the present study, minor unwanted effects were reported by some participants, including temporary fatigue and arm stiffness/numbness. While these unwanted effects resolved within the hour following the intervention, it may be advised to closely monitor the level of exertion and other factors such as stiffness or pain in future intervention studies.

Main limitations of this study include a small sample size and inherent limitations of single subject designs. This work, however, was an essential step toward determining the feasibility of the intervention in post-stroke participants having different levels of motor recovery, before larger clinical trial can be undertaken. Although we did not have a no-treatment or standard treatment control group, this limitation was partially addressed with the use of an AABA design, controlling for the passage of time and ensuring that participants were stable prior to the beginning of the intervention. However, whether the improvements are specifically related to the musical aspect of the intervention cannot be determined. Nevertheless, this preliminary study provides evidence that an intervention providing musical feedback that combines intensive practice and high motivation can be beneficial for chronic stroke survivors. A future larger study will also allow us to compare the intervention to a standard treatment. Future directions for research include the investigation of a larger pool of participants, possibly with longer training duration and a longer-follow-up.

## Conclusion

This study provides the first evidence that a piano training intervention combined to home practice can lead to improvements in manual dexterity, finger movement coordination, and functional use of the upper extremity that persist 3 weeks after the intervention. In addition to representing a socially valued and enjoyable activity, piano training has the potential to be self-managed and to enable people with chronic stroke to pursue upper extremity exercises beyond the usual rehabilitation time frame.

## Conflict of Interest Statement

The authors declare that the research was conducted in the absence of any commercial or financial relationships that could be construed as a potential conflict of interest.

## Abbreviations

BBT, Box and Block Test; CMSA, Chedoke McMaster Stroke Assessment; FTN, Finger to Nose Test; FTT, Finger Tapping Test; Jebsen, Jebsen Hand Function Test; MST, Music-supported Therapy; NHPT, Nine Hole Peg Test.
